# The Treatment of Uterine Myomata

**Published:** 1898-11-26

**Authors:** Arthur E. Giles

**Affiliations:** Assistant Surgeon, Chelsea Hospital for Women.


### Hospital Clinics and Medical Progress.

### THE TREATMENT OF UTERINE MYOMATA.

By Arthur E. Giles, M.D., B.Sc., F.R.C.S., Assistant Surgeon, Chelsea Hospital for Women.

**What Becomes of Fibroid Tumours of the Uterus when left Alone?**—This is one of the most important questions in relation to the treatment of these tumours, for to many men the answer to it is the crux of the whole matter. The theoretical conservative at one extreme says that fibroids never destroy life, and that consequently no fibroid should be operated upon. The theoretical opposite extremist maintains that every tumour menaces the woman's life, and that her danger is so imminent that every case ought to be operated upon as soon as possible. Personally, I do not believe that the representative of either of these extremes exists, although discussions on the subject would sometimes lead one to infer that all gynæcologists hold either the one or the other view. For I know men of reputed conservative tendencies who occasionally advise and practise operation; and others, credited with the *cacœthes operandi,* who interfere in only a small proportion of the cases they see. The inference is, of course, that the former consider that fibroids threaten life in some cases, and that the latter trust to a certain number of tumours to give no trouble. This brings us to the position of the moderate man, namely, that in a proportion of cases myomata may be safely left alone; whilst in the remainder they will need surgical intervention. I am prepared to affirm that all the gynæcologists I know are moderates with variations; their differences turning on the ratio of frequency of the two classes of myomata. What is this ratio? That is precisely the point that is not known; if it were, there would be much less argument about the justifiability of operations for fibroids. But if this point must at present remain open, there are others which admit of definite replies to the question which heads the paragraph. In considering the fate of fibroids it is necessary to deal separately with the three main classes of these tumours, for they behave somewhat differently:—

1. Submucous Myomata: The only favourable termination is the formation of a polypus, and its extrusion. Occasionally, this may occur spontaneously, but much blood may be lost before the process is complete. The unfavourable courses are hæmorrhage leading to the undermining of health; and septic infection, either primary, or following twisting of the pedicle and gangrene.

2. Subserous Myomata: These may terminate favourably by simply ceasing to grow, or by becoming calcified. They may take an unfavourable course by undergoing fibrocystic (mucoid) degeneration, by becoming infected, or by attaining such a size as to lead to injurious pressure on surrounding organs. In the event of pregnancy supervening there is the added risk of obstructed labour with all its dangers.

3. Interstitial Myomata: These combine the characteristics of the other two forms, in regard to both their favourable and unfavourable issues. In the former direction they may become polypoid and be spontaneously extruded, or they may cease to grow after calcification or otherwise, or they may even undergo spontaneous involution and reduction in size, especially after childbirth. Their unfavourable courses are hæmorrhage and septic infection, as may be the case with submucous myomata; or, as with the subserous kind, fibro-cystic degeneration, interference with pregnancy or labour, and injurious pressure effects. "Intestinal obstruction may result from pressure on the rectum, or a loop of small bowel may become entangled by the stalk of a pedunculated myoma. Pressure on urethra or ureters may lead to cystitis, sacculated kidneys, nephritis, or hydronephrosis" (Sutton and Giles, "Diseases of Women," p. 191).

These various results may be stated in tabulated form:—

| Variety. | Favourable Results. | Unfavourable Results. |
|---|---|---|
| Submucous | Polypoid formation and extrusion. | Hæmorrhage.<br>Septic infection. |
| Interstitial | Polypoid formation and extrusion.<br>Cessation of growth.<br>Calcification.<br>Involution, spontaneous or after childbirth. | Hæmorrhage.<br>Septic infection.<br>Fibrocystic degeneration.<br>Pressure effects.<br>Production of abortion.<br>Interference with labour. |
| Subserous | Cessation of growth.<br>Calcification. | Septic infection.<br>Fibrocystic degeneration.<br>Pressure effects.<br>Interference with labour. |

It will be seen that the unfavourable possibilities outnumber the favourable ones; and I believe that if the history of every case of myoma could be traced it would be found that it is only in a small minority of cases that a spontaneous favourable result occurs. I have heard the argument put in the following form: "What is the mortality of fibroids when left alone? Perhaps 1 in 50, say 2 per cent. What is the mortality of hysterectomy? From 10 to 30 per cent., or even higher, according to the operator. Ergo: All fibroids should be left alone." This form of argument is, of course, based on a conspicuous fallacy. The fact is that we do not know in the least what is the mortality of fibroids if left alone, for the simple reason that nowadays when they give rise to serious trouble they are operated upon; those that escape operation are, in the main, those that have taken a beneficent course. If operation is undertaken because of severe hæmorrhage, septic infection, or serious pressure effects, and the patient dies, the death is transferred from the account of fibroid tumours to that of operation for the same.

This remark does not, of course, apply to the preoperative days of fibroids; but I do not know of any statistics of their mortality in those days; and it is probable that in many instances they were not diagnosed, for we know how difficult the diagnosis is in some cases at the present time.

**When Ought Fibroid Tumours of the Uterus to be Operated upon?**—If the facts as stated above be accepted, it will be granted that a large proportion of fibroid tumours require surgical treatment sooner or later; and the only question is, When? To narrow down the discussion of this point, it is convenient to recognise three stages in the progress of a fibroid tumour: (*a*) The stage in which it causes only slight disturbance, or none at all; (*b*) the stage in which it interferes with health and usefulness, but does not threaten life; and (*c*) the stage in which life is endangered. This division shows where the shoe pinches. Few, if any, surgeons would interfere in the first stage; few, if any, would leave a woman to die unrelieved in the third stage. To my mind, at any rate, the rule of practice is quite clear: In the first stage do not operate, but watch; whilst, according to the predilection of the medical man, drugs or electricity may be tried, or, better still, we may leave the patient alone. In the third stage, operate without delay.

It is in the second stage that difficult questions arise. When confronted with any given case, and asked, "What do you advise?" one's answer must depend on circumstances. We cannot tell whether that particular tumour is going to settle down without serious troubles or not; with the chance of its doing so, we naturally hesitate to advise operation with its serious risk to life; on the other hand, with the chance of the eventual necessity for operation we hesitate to allow the patient to suff:r pain, loss of health, and diminished usefulness, while we are merely postponing operation, possibly to a time when it will have to be performed under less favourable circumstances. Some surgeons will advise waiting for the menopause, saying that fibroids often diminish in size when that time comes. True; but the patient may be under 40 years of age, and we know that when a myoma is present the menopause is often delayed, perhaps till the age of 50. Is she to suffer for ten years? Moreover, in many cases the menopause does not bring the expected relief. The tumour, left alone, will probably not cause death; but it is too often not realised that death is not the only evil which the patient has to fear. She may have to earn her living, and incapacity for this may mean pauperism or starvation; suffering may demand surgical help, just as much as threatened life; nor are the risks spoken of above to be overlooked. Clearly this is no matter for dogmatism and extremes, but rather for open-minded caution. The following is, perhaps, the safest line of treatment. If symptoms be not very severe, the patient should be kept under observation. If there be much hæmorrhage an attempt may be made to deal with it by hæmostatic drugs, by the application of electricity (positive pole in the uterus), or by intra-uterine exploration in case a polypus is to blame. If the patient can afford to be an invalid for a time prolonged rest may be tried. If she has to earn her daily bread, and her condition prevents her from doing so; if severe hæmorrhage persists in spite of palliative measures; if urgent pressure-symptoms arise or other grave complications threaten, then the question of operation should be raised; and if, after the facts have been placed before the patient and the dangers pointed out to her, she elects to take the risk for the chance of cure, I think we are bound to give her that chance and operate. Space will not allow of a detailed review of the different operative procedures, such as ligature of the uterine arteries, oöphorectomy, myomectomy, supravaginal hysterectomy, and pan-hysterectomy; but I believe that in most instances the most satisfactory measure is supravaginal hysterectomy.

